# Novel Generation‐Skipping Inheritance Pattern of Marfan Syndrome Due to *FBN1* Insertional Translocation: Diagnostic Utility of FISH and Implications for Genetic Counseling

**DOI:** 10.1155/crig/1611720

**Published:** 2026-03-09

**Authors:** Breanna Beers, Hamilton Wexler, Gretchen MacCarrick

**Affiliations:** ^1^ National Human Genome Research Institute, Bethesda, Maryland, USA, genome.gov; ^2^ Bloomberg School of Public Health, Johns Hopkins University, Baltimore, Maryland, USA, jhu.edu; ^3^ Mckusick Nathan Department of Genetic Medicine, Johns Hopkins University School of Medicine, Baltimore, Maryland, USA, jhu.edu

**Keywords:** insertional translocation, Marfan

## Abstract

Marfan syndrome (MFS) is an autosomal dominant connective tissue disorder caused by pathogenic variants in the fibrillin‐1 (*FBN1*) gene on Chromosome 15q21.1. A 3‐year‐old female presented to the clinic with MFS and a family history of an affected maternal uncle and maternal great‐aunt. The proband and the uncle had a positive thoracic aortic aneurysm and dissection (TAAD) panel for MFS revealing an *FBN1* deletion. This was confirmed on proband’s chromosome microarray; however, the mother was negative for the *FBN1* deletion. Fluorescence in situ hybridization (FISH) was used in this case to show a unique chromosome rearrangement in the unaffected mother with an insertional translocation of the 15q21.1 loci (*FBN1*) to Chromosome 7p. This led to an affected child who inherited the nontranslocated Chromosome 7 and the 15q21 (*FBN1*) deletion. Thus, individuals in the family inheriting Chromosome 7 with the *FBN1* insertional translocation are protected from the MFS phenotype. This supports the known autosomal dominant inheritance pattern while allowing for uncharacteristic skipping of generations of MFS in this family.

## 1. Introduction

First described in 1896, Marfan syndrome (MFS) is an autosomal dominant connective tissue disorder with multisystem involvement caused by pathogenic variants in *FBN1*, which encodes fibrillin‐1. Loss‐of‐function variants in *FBN1* are hypothesized to result in disordered microfibril matrices, weakened fibrillar elastic matrix, and altered transforming growth factor‐beta (TGFB) signaling [1, 2]. The clinical and genetic factors that characterize MFS are aggregated within the revised 2010 Ghent criteria for diagnosis [3]. The three key clinical indicators include thoracic aortic aneurysm and dissection (TAAD), ectopia lentis, and characteristic musculoskeletal features including scoliosis, pectus anomalies, and arachnodactyly. Genetic indicators include a pathogenic variant in *FBN1* or a first‐degree relative with MFS [3].

About 75% of pathogenic *FBN1* variants are inherited from an affected parent, leaving 25% apparently *de novo*, although rare cases of parental mosaicism have been reported [4]. The most common molecular workup for suspected MFS is a multigene panel for conditions associated with TAAD, although single‐gene testing of *FBN1* may be considered when the clinical suspicion is highly specific. Deletion/duplication analysis should be included, as about 5% of pathogenic variants in *FBN1* are copy number variants [4].

The spectrum of pathogenic variations in *FBN1* includes missense, frameshift, splice site variants, and both intragenic deletions and chromosomal deletions [4]. Pathogenic translocations have been reported in the literature when the translocation causes a breakpoint in *FBN1*, including a report of a balanced/reciprocal translocation 46,XX, t(2; 15)(q22; q21.1) where fluorescence in situ hybridization (FISH) and optical genome mapping (OGM) were performed, ultimately refining the breakpoint to within Intron 55 of *FBN1,* suggesting a minor loss of approximately 2.2 Kb on Chromosome 15 [5, 6].

Here, we report a familial case of MFS where an insertional translocation involving the *FBN1* locus was passed down in an unbalanced manner, explaining an atypical inheritance pattern of skipped generations within a family affected by MFS and demonstrating the need for cytogenomic techniques to clarify clinical observations.

## 2. Case Presentation

A three‐year‐old female with a diagnosis of MFS presented to the Cardiovascular Connective Tissue Disorders clinic at Johns Hopkins University. Prior to this visit, her mother sought evaluation at an outside genetics clinic for suspected MFS based on features of hypotonia, flat feet, mild pectus excavatum, mild developmental delay, and long, thin fingers. The echocardiogram showed a mildly dilated aortic root and mild mitral valve prolapse with trivial mitral regurgitation.

**FIGURE 1:**
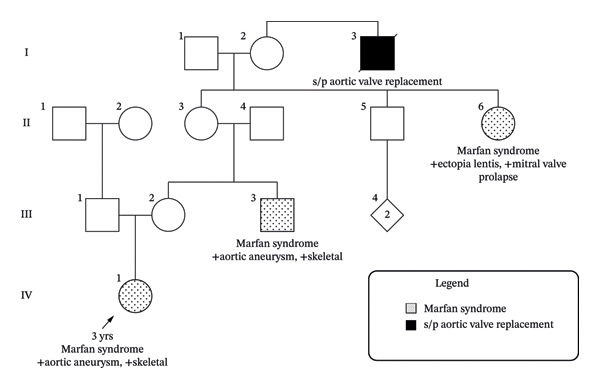
Pedigree showing skipped generations of Marfan syndrome.

Family history included a maternal uncle and maternal great‐aunt who were both clinically diagnosed with MFS (Figure 1). The maternal uncle was found to have a heterozygous pathogenic 15q21 deletion on TAAD gene panel testing. His mother, the proband’s maternal grandmother, tested negative for this deletion and appeared clinically unaffected. The maternal great‐aunt has not undergone genetic testing, despite being clinically affected.

**FIGURE 2:**
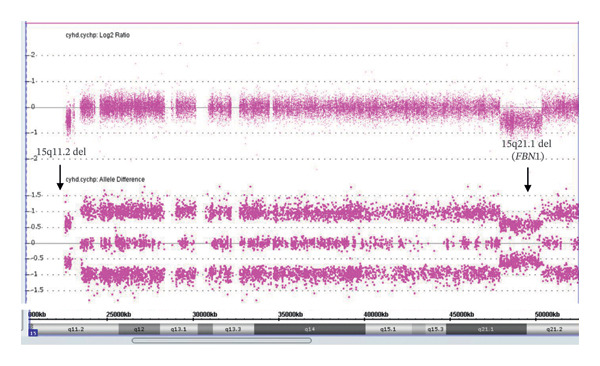
Affymetrix array showing deletion on 15q11.2 and 15q21.1q21.2 (*FBN1*) in the proband.

The TAAD panel and chromosome microarray (CMA) performed at the outside clinic on the 3‐year‐old proband confirmed a multigene deletion involving *FBN1* and defined the breakpoints to containing 18 genes across the 2448 Kb deletion from 15q21.1 to 15q21.2 (Figure 2). The CMA results (including maternal array follow‐up) on the proband were arr[GRCh37] 15q21.1q21.2 (48108118_50556137)x1 causing a pathogenic deletion of 2448 Kb and arr[GRCh37] 15q11.2(22770421_2328905)x1 mat causing a likely pathogenic deletion ( low penetrance) of 512 Kb. Four genes within the 15q21.1–15q21.2 region (most notably *FBN1* but also including *SLC24A5, SLC12A1,*and *CEP152*) are associated with known clinical disorders at present. The other three genes are associated with autosomal recessive conditions (oculocutaneous albinism Type VI, Bartter syndrome, and primary microcephaly), and our proband showed no features of these disorders, so no secondary sequencing of the other alleles was pursued.

CMA also indicated a loss of 15q11.2, a region containing two low‐copy repeats (regions of repetitive DNA which are susceptible to rearrangements) known as Breakpoint 1 and 2 (bp1 and bp2), and four genes: *NIPA1, NIPA2, CYFIP1,* and *TUBGCP5*. The pathogenicity of this deletion and its impact on our proband is unclear, as it has been associated with developmental delay, speech impairment, and learning or behavioral challenges but is frequently inherited from an unaffected parent [7]. This deletion has low penetrance, and the expression of any associated phenotype has been estimated to be between 8% and 10%. This deletion is thus consistent with being a susceptibility locus for neurodevelopmental phenotypes. Our patient had minor motor delays likely related to the MFS diagnosis but no other neurodevelopmental delay.

The proband’s mother was negative for the 15q21 (*FBN1*) deletion but was positive for the 15q11.2 deletion. The proband’s father was not available for testing. However, the 15q21 (*FBN1*) deletion—present in the proband but absent in the mother—appeared to be the same familial deletion present in the maternal uncle and absent in the mother.

**FIGURE 3:**
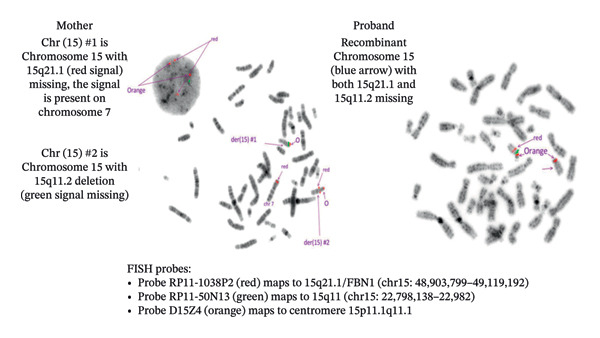
FISH probes showing (1) the mother with 15q21.1 (*FBN1*) signal inserted on Chromosome 7 and a deletion of 15q11.2, and (2) the proband with a deletion of 15q21.1 (*FBN1*) and 15q11.2 on the same Chromosome 15.

To elucidate the unusual inheritance pattern in this family, targeted FISH was pursued (Figure 3). The cytogenomic results of the proband were as follows: arr[GRCh37] 15q21.1q21.2(48108118_50556137)x1; a pathogenic deletion of 2248 Kb and arr[GRCh37] 15q11.2(22770421_23282905)x1 mat; likely a pathogenic variant, a low‐penetrance deletion of 512 Kb. Probes utilized for FISH included the following: Probe RP11‐1038P2 that maps to 15q21.1 (chr15:48,903,799–49,119,192) and is labeled in red in Figure 3, and Probe RP11‐50N13 that maps to 15q11 (chr15:22,798,138–22,982) and is labeled in green in Figure 3. Additionally, centromere probes (orange in Figure 3) of Chromosome 15 (D15Z4) and Chromosome 7 (DZ71) were used in analysis for the mother. The cytogenomic results of the mother were as follows: ish ins(7; 15)(p1?5;q21.1q21.2)(RP11‐1038P23+,D7Z1+;RP11‐1038P23+), del(15)(q11.2q11.2)(RP11‐50dtN13−,RP11‐1038P23+), del (15)(q21.1q21.2)(RP11‐50N13+,RP11‐1038P23−).

Thus, FISH analysis revealed a balanced insertion in the unaffected mother, with one 15q21 locus translocated to Chromosome 7p. As a result, one Chromosome 7 carried material from 15q21 (including the *FBN1* region), while one Chromosome 15 lacked this segment. The same Chromosome 15 harbored the inherited 15q11.2 deletion (Figure 3).

**FIGURE 4:**
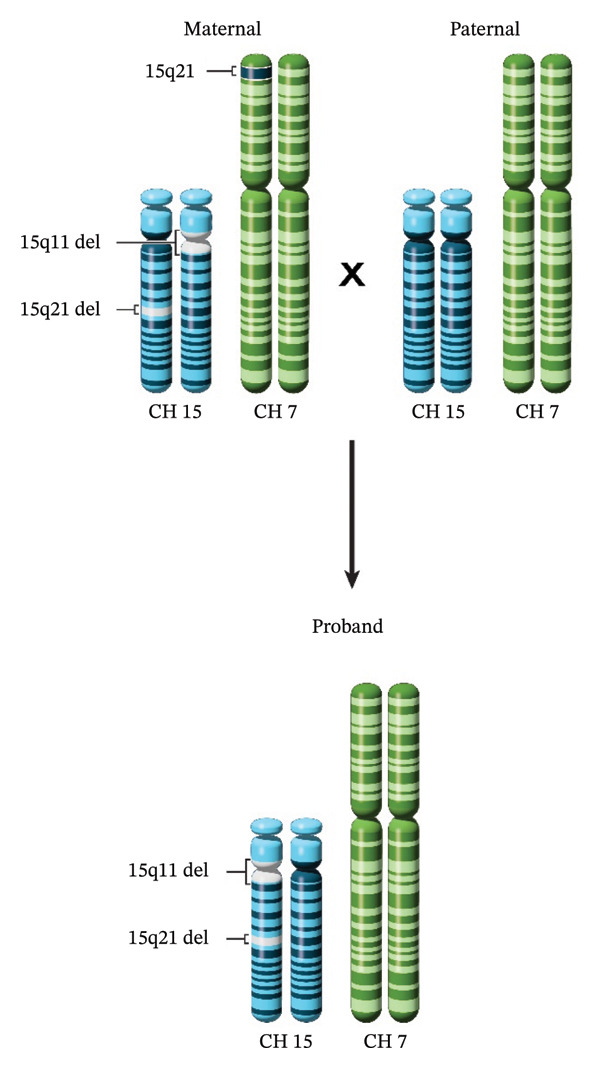
Ideogram of chromosomes in the proband and parents. The mother carries the insertional translocation with 15q21.1 (*FBN1*) translocated to Chromosome 7, thus has two copies of the *FBN1* gene and is unaffected with Marfan syndrome. The proband has inherited the 15q21.1 (*FBN1*) deletion and the nontranslocated Chromosome 7, thus has one copy of *FBN1* and has Marfan syndrome. Both generations have the low‐penetrant 15q11.2 deletion.

This insertional translocation explains the unusual inheritance pattern observed in this family. Among family members who inherit the 15q21 (*FBN1*) deletion, those who inherit the Chromosome 7 containing the insertional translocation are protected from the MFS phenotype, since they have two functional copies of *FBN1*. However, they can have children who inherit the 15q21 (*FBN1*) deletion with the nontranslocated Chromosome 7, resulting in one copy of *FBN1* and MFS. These children, including our proband, present with familial MFS despite having apparently unaffected parents (Figure 4).

The proband is currently under standard ophthalmologic and cardiovascular treatment protocols for MFS. Her parents received genetic counseling regarding recurrence risks based on these genetic testing results. The maternal uncle declined further cytogenomic testing. Patient consent was obtained for the case report.

## 3. Discussion

This case highlights the diagnostic and counseling challenges associated with atypical inheritance patterns, particularly in the context of an insertional translocation involving the *FBN1* locus.

The proband exhibited classic clinical features of MFS, yet the inheritance pattern, described as skipped generations, required cytogenomic tools to clarify. While MFS classically follows an autosomal dominant pattern due to pathogenic variants in the *FBN1* gene, this case reveals an atypical inheritance linked to a structural chromosomal rearrangement.

In terms of recurrence risk, when a parent carries a balanced interchromosomal or intrachromosomal insertion, there is a theoretical risk of a 50% combined recurrence risk for monosomic or trisomic imbalance in an offspring [8]. However, this is likely impacted by the chromosomal region and the risk for possible miscarriage due to imbalances. A small study of interchromosomal insertions (ITs) by Van Hemel and Eussen reports a genetic risk of monosomy or trisomy for IT carriers in the 32%–36% range [8].

Genetic counseling must include detailed discussion of possible reproductive outcomes, including the risk of producing offspring with (1) MFS due to an unbalanced insertional translocation, (2) trisomy of 15q21 (*FBN1*) which has an unreported phenotype, or (3) balanced chromosomes. Options such as preimplantation genetic testing (PGT) and prenatal diagnostic testing should be presented to the family for consideration in future pregnancies. Furthermore, asymptomatic siblings should undergo genetic testing to assess their carrier status of a balanced insertional translocation, as this may impact their own reproductive planning and the risk of having a child with MFS.

In our specific family, we infer that the mother (III‐2), maternal grandmother (II‐3), and maternal great‐grandmother (I‐2) all have the 15q21 *FBN1* insertional translocation, as well as a great‐great grandparent. It would be interesting to follow familial segregation, especially in the maternal uncle (II‐5), to determine if there are any sex selection differences between men and women because all of our IT carriers are females.

This case also underscores the limitations of traditional genetic testing methods, such as targeted single‐gene or multigene panels, in detecting complex chromosomal rearrangements associated with rare diseases. Advanced cytogenomic techniques will continue to play a pivotal role in elucidating inheritance mechanisms not detected by traditional sequencing methods. The possibility of a chromosomal rearrangement should be considered whenever a large deletion or duplication is detected, especially in known syndromes presenting with atypical inheritance patterns.

## Funding

No funding was received for this manuscript.

## Disclosure

This abstract was presented at the American College of Medical Genetics 2025 Annual Education Conference, and the abstract was published online through Genetics in Medicine Open, Volume 3, Supplement 2, 1032912025 Open Access. P424: Novel inheritance pattern of Marfan syndrome due to *FBN1* translocation: Diagnostic utility of FISH and implications for genetic counseling.

## Conflicts of Interest

The authors declare no conflicts of interest.

## Data Availability

Data sharing is not applicable to this article as no datasets were generated or analyzed during the current study.
